# Epithelioid angiomyolipoma with tumor thrombus in IVC and right atrium

**DOI:** 10.4322/acr.2020.190

**Published:** 2020-09-02

**Authors:** Deepika Gupta, Vikarn Vishwajeet, Himanshu Pandey, Mahendra Singh, Binit Sureka, Poonam Elhence

**Affiliations:** a All India Institute of Medical Sciences, Department of Pathology. Jodhpur, India.; b All India Institute of Medical Sciences, Department of Urology. Jodhpur, India.; c All India Institute of Medical Sciences, Department of Diagnostic and Interventional Radiology. Jodhpur, India.

**Keywords:** Kidney, Angiomyolipoma, Histology, Epithelioid Cells, Immunohistochemistry

## Abstract

Epithelioid angiomyolipoma is an uncommon subtype of renal angiomyolipoma associated with potentially malignant behavior and is considered a distinct entity by the World Health Organization classification of renal tumors. We present a case of an epithelioid variant of angiomyolipoma with extension into the renal vein, inferior vena cava reaching up to the right atrium. Pre-operatively, a diagnosis of renal cell carcinoma was considered based on imaging findings. Intra-operatively due to extensive adhesions, surgical resection was not performed and only tissue sampling was performed for histopathology. Microscopic examination revealed short fascicles of spindle cells and perivascular epithelioid cells. A differential diagnosis of renal cell carcinoma with sarcomatoid differentiation was considered. The immunohistochemical profile showed tumor cells that express Melan-A and smooth muscle actin, while they were negative for pan-cytokeratin, PAX8, CK7, CD117 and CD34. Therefore a diagnosis of epithelioid angiomyolipoma was rendered. The presence of intravascular thrombi on radiological investigation and carcinoma-like growth pattern on light microscopy may compound an erroneous diagnosis of renal cell carcinoma. Hence, it is prudent for the urologist to consider differential diagnosis other than renal cell carcinoma when confronted with a renal neoplasm presenting with intravascular thrombi. In these cases, a core biopsy should be planned pre-operatively and diagnosis should be made with aid of appropriate immunohistochemical markers.

## INTRODUCTION

Angiomyolipoma (AML) is a rare hamartomatous tumor, which usually arises from the visceral organs, mainly in the kidney, lung and liver. AML is composed of an admixture of mature fat, smooth muscle and blood vessels.^1^ Apart from the classical AMLs, the recent WHO classification describes several morphological variants. These include AML with epithelial cysts, oncocytoma-like AMLs, microscopic AMLs (microhamartoma) and intraglomerular lesions. Epithelioid AMLs (EAML) is described under a separate heading in the current WHO classification of tumor and is also known as PEComa of the kidney (perivascular epithelioid cell tumors).^2^ Most cases of AMLs occur sporadically and only a few of them (<10%) are associated with tuberous sclerosis.^3^ Although they are benign lesions, larger tumors particularly epithelioid variant can, behave aggressively and may have extra-renal extension.^4^ Extension of an AML into the renal veins, inferior vena cava (IVC) and heart is rare, unlike renal cell carcinomas.^5^ ^,^ ^6^ We report a rare case of a 40-year-old woman who presented with a large AML of the right kidney, with extension into the renal vein and IVC, up to the right atrium.

## CASE REPORT

**Figure 1:**
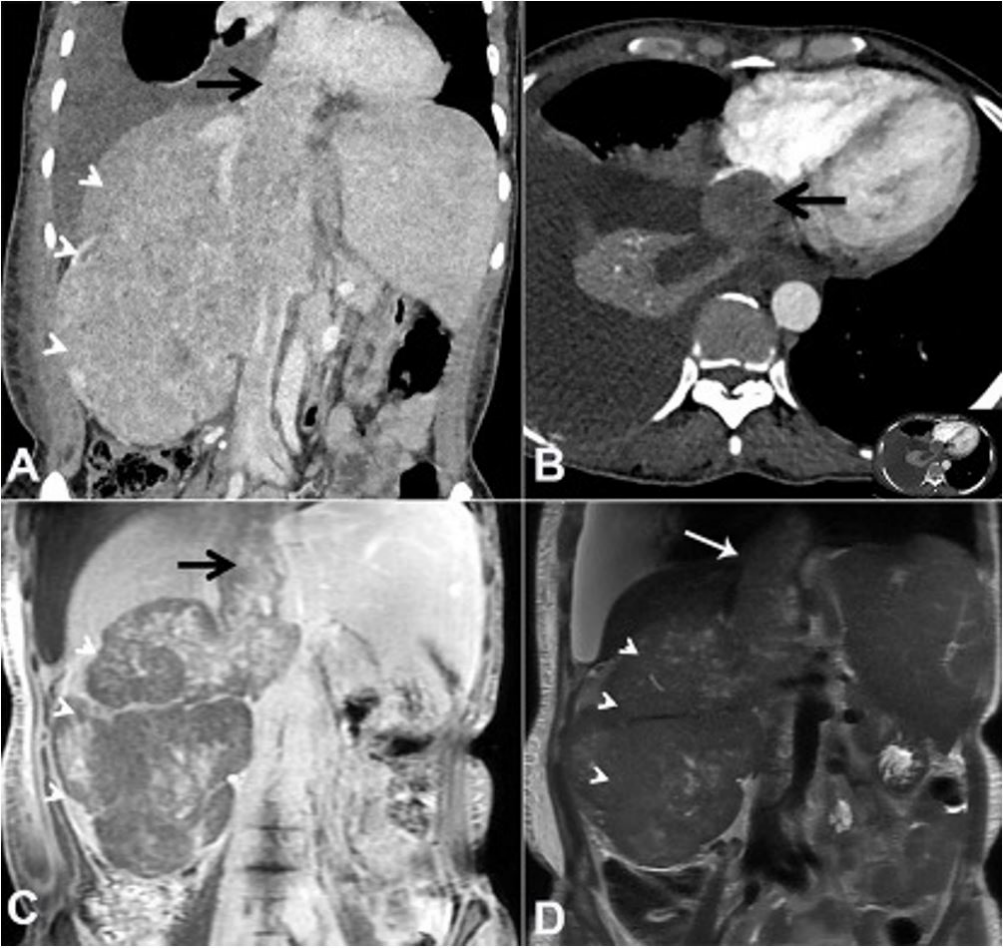
**A –** contrast-enhanced abdominal CT, Coronal plane, showing heterogeneously enhancing mass lesion (arrowheads) almost replacing the right kidney with tumor thrombus (arrow) extending into the right renal vein and IVC; **B –** axial plane showing a tumor mass within the right atrial chamber (arrow) (inset represents the whole slice); **C –** Coronal T1-weighted post-contrast abdominal MRI showing patchy moderate central enhancement (arrowheads) with enhancement of thrombus (arrow) expanding the IVC; **D –** Coronal T2-weighted abdominal MRI showing relatively isointense mass lesion (arrowheads) with interspersed hyperintense areas consistent with necrosis.

A 40-year old female presented with complaints of abdominal pain, predominantly on the right side, which gradually worsened along with occasional episodes of vomiting over 2 months. She also complained of a vague lump in the abdomen. Physical examination revealed respiratory distress, hypotension and bilateral pedal edema. On abdominal examination, a 15x15x10 cm lump was bimanually palpable over the right hypochondrium, epigastrium and the right lumbar region, which had a hard consistency and did not move with respiration. Right renal angle fullness was present. The routine investigations showed anemia, thrombocytopenia, hyperkalemia, and hyponatremia. The chest X-ray confirmed the presence of right-sided pleural effusion. The computed urotomography depicted a large heterogeneously enhancing mass with internal non-enhancing cystic to necrotic areas, measuring 10.5x11.9x16.0 cm in the right kidney. The lesion was invading and expanding into the renal vein, intrahepatic, suprahepatic IVC and reached up to the right atrium (Figure 1).

Possibility of a right renal cell carcinoma (RCC) with tumor thrombus into renal vein and IVC with wall invasion was considered. In view of radiological diagnosis of RCC, the patient was planned for an exploratory laparotomy. On the exploratory laparotomy, dense adhesions were present between the tumor, colon and infrarenal as well as suprarenal IVC. No dissection plane was found between IVC and the tumor.

**Figure 2:**
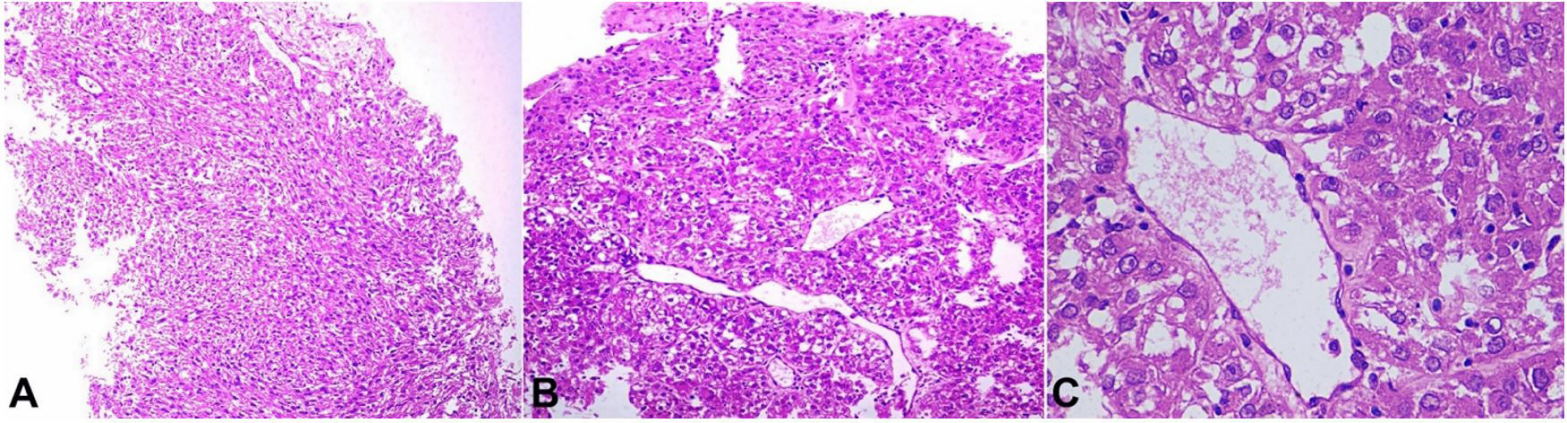
Photomicrograph of the biopsy showing in **A –** variable morphology with fascicular arrangement (H&E, 100X); **B –** characteristic perivascular clusters of tumor cells (H&E, 100X); **C –** tumor cells with epithelioid morphology (H&E, 400X).

Therefore, in view of tumor unresectability, a biopsy was taken and the resection was suspended. The microscopic examination showed a varied morphology with short fascicular arrangement of spindle shaped tumor cells, with intervening thin walled vascular channels (Figure 2A), and nests and lobules of tumor cells with epithelioid morphology. These epithelioid tumor cells appeared to be more centered on dilated thin walled vascular channels (Figure 2B). The tumor cells showed moderate nuclear pleomorphism with vesicular chromatin, prominent nucleoli and moderate to abundant amounts of clear to pale eosinophilic cytoplasm (Figure 2C).

**Figure 3:**
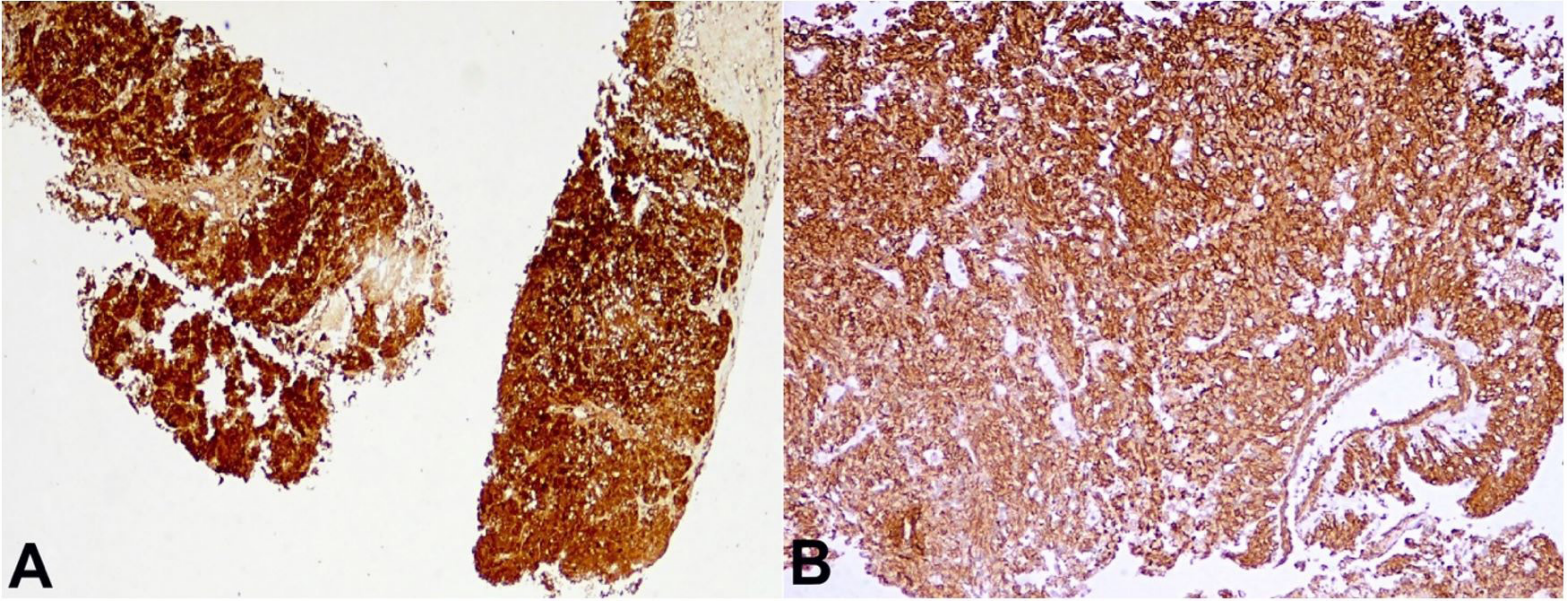
Photomicrograph of the biopsy showing the tumor cells show strong immunoreactivity for Melan A (**A –** 40X) and SMA (**B –** 100X).

Scattered mitotic figures and a few interspersed multinucleated tumor cells were also seen. A possibility of RCC with sarcomatoid differentiation was considered. However, on immunohistochemistry, the tumor cells showed immunoreactivity for Melan A (Figure 3A) and SMA (Figure 3B) and were negative for PAX-8, Pan-Cytokeratin, Myogenin, CD117, CD34, CK7, PAX-8 and HMB-45.

In view of classical morphology and supporting immunohistochemistry findings, a diagnosis of EAML was considered. Preoperative radiological diagnostic consideration of RCC was excluded by appropriate immunohistochemical results. The patient was discharged after appropriate supportive care and counselling. Once the histopathological diagnosis was made, the case was discussed at a multi-disciplinary team meeting, and possible treatment modalities were discussed. Subsequently, a telephonic conversation with the patient’s relative was made. However, the patient refused to come to the hospital for any treatment. Finally, the patient succumbed to her illness after 2 and half months.

## DISCUSSION

PEComas represent mesenchymal tumors characterized by unique perivascular epithelioid cells expressing both melanocytic and myoid markers. They can occur at any anatomical sites, with particular predilection for visceral location such as kidney, liver and lung. PEComas of the kidney encompass classic AML and its histological variant (AMLs with epithelial cysts, oncocytoma-like AMLs, microscopic AMLs and intraglomerular lesions and epithelioid AMLs).^1^ AMLs represent 0.3-3% of all renal tumors, with a female preponderance, due to hormonal influences.^7^ They can range from microscopic lesions to very large tumors with extension into IVC and heart.^6^ Most of these tumors are asymptomatic and detected incidentally. Larger tumors (>4 cm) can be symptomatic with flank pain and hematuria, or following retroperitoneal hemorrhage from intra-tumoral vessels.^8^

The classic renal AML is a benign solid tumor which is typically composed of dysmorphic blood vessels, smooth muscle cells, and mature adipose tissue. These can show predominance of smooth muscle elements or adipose tissue, depending on which it can be labelled as leiomyoma-like or lipoma-like.^2^ In a recent study by Çalışkan et al.,^9^ the authors described 28 cases of renal AMLs and classified in three categories: fat-rich (82.1%), fat-poor (14.3%) and epithelioid (3.6%). In the study by Aydin et al.,^3^ classic AMLs accounted for 76.8% cases while epithelioid variants, epithelial cysts and microscopic AMLs were noted in 7.7% cases, 6.7% cases and 10.8% cases, respectively. EAMLs can be aggressive, with local extension, distant metastasis, higher recurrence and mortality.^5^ ^,^ ^10^ It was first described in 1997 by Eble et al.^11^ and is composed of epithelioid cells, polygonal cells, varying degrees of nuclear atypia, with little or no fat cells. According to recent articles, EAMLs can specifically be categorized into typical and atypical types, and the atypical one possesses aggressive behavior.^12^ The malignant potential of EAMLs is unequivocally demonstrated in literature. A few studies have analysed several clinico-pathologic factors for prognosticating EAML patients. Nese et al.^13^ proposed a prognostic risk category for EAML cases by including five adverse parameters: EAMLs with TSC and/or coexisting classical AML, tumor size more than 7cm, presence of a carcinoma-like growth pattern, perirenal fat extension and/or renal vein involvement and necrosis. Tumors possessing 0-1 parameter, 2-3 parameters and 4 or more parameters were stratified into low risk, intermediate risk and high-risk categories, respectively. Among these groups, disease progression risks were 15%, 64% and 100%. Another study suggested presence of at least three out of four parameters (atypical epithelioid cells ≥ 70%, mitotic figures ≥2/10hpf, atypical mitotic figures and necrosis) to differentiate benign EAMLs with atypia from malignant EAMLs with atypia.^12^ The present case had several features including extrarenal extension, renal vein involvement, mitotic figures and carcinoma-like growth pattern, suggesting malignant behavior.

Classic AMLs, particularly the larger ones, can rarely have involvement of the renal vein or IVC. This might be attributed to multifocal genesis of tumor, instead of direct vascular involvement.^14^ The first case of renal AML with IVC was reported by Kutcher et al.,^15^ and the first case of renal AML with extension to heart was reported by Rothenberg et al.^16^ Riviere et al.,^6^ in their review, found that among patients with AML, 44 had IVC extension and most of them had large tumors (>4 cm) at presentation and more than 67% of patients were symptomatic. The median age of presentation was 46.6 years, and only seven patients had right atrial extension and all of them were female.

The current diagnostic methods include ultrasound, Computed Tomography (CT) and Magnetic Resonance Imaging (MRI). Because of its fat component, the preferred diagnostic method for AML is CT.^17^ The fat content appears as hypodensity on CT and as a hyperechoic signal on sonography.^18^ Approximately 5% of AMLs lack fat and, therefore, cannot be differentiated from RCCs. Due to their peculiar characteristics, EAMLs resemble conventional RCC either histologically and radiologically, and have similar cytologic features on fine-needle aspiration.^19^ EAMLs and RCC both frequently present with vague flank pain, a palpable mass or hematuria.^20^ The definitive method for the differential diagnosis between EAMLs and RCC is based on immunohistochemical markers. RCCs are immunoreactive for PAX-8, cytokeratin and EMA, which are negative in EAMLs. By contrast, EAMLs show co-expression of melanocytic markers (HMB-45 and melan-A) and myoid markers (SMA, MSA, calponin and/or desmin), which are not found in RCC.^12^ However, one of the melanocytic marker, HMB-45, was negative in the present case. Although this is an infrequent finding, it is well reported in the literature. Aydin et al^3^, reported HMB-45 and Melan A positivity in 92% and 80% cases of EAMLs, respectively. In their study, expression of any one of these markers were noted in 100% cases. Isolated cases showing loss of HMB-45 expression in AML are also reported by Hohensee et al.^21^ and Lin et al.^22^ Hohensee et al,^21^ in their case, reported absence of both melanocytic markers on IHC in a case of renal EAML. Diagnosis was confirmed by presence of premelanosomes on electron microscopy examination. The authors ascribed lack of IHC expression for these antibodies to aberrant antigen expression in tumor tissue.

Currently, there is no standard treatment for renal AML. Annual imaging examinations are proposed for patients with sporadic tumors measuring <4 cm. However, for large tumors (>4 cm), the majority of previous studies recommend surgical treatment. For tumor thrombus involving renal vessels, the inferior vena cava, and even the right atrium, a thrombectomy is reasonable. In contrast to conventional RCC, EAMLs are sensitive to chemotherapy because they are part of the perivascular epithelioid cell tumor group. EAMLs have been reported to respond to doxorubicin.^10^

In conclusion, EAML is an uncommon variant of AML and is a close mimicker of renal cell carcinoma, particularly when there is intravascular spread of tumor cells in EAML. Pathologically, a carcinoma-like growth pattern in absence of adipocytic components may further add to erroneous diagnosis. Though rare, it is prudent for the treating surgeon to consider differential diagnosis other than renal cell carcinoma when confronted with a renal neoplasm presenting with intravascular thrombi. In these cases, a core biopsy should be planned pre-operatively and diagnosis should be made with aid of appropriate immunohistochemical markers.
